# 

**Published:** 1888-06-23

**Authors:** 


CONTENTS.

**Anonymous Slanderers and Working Men** - 189

**The Old and the New.**—Lines Written in Winchester Hospital - 190

**Patients' Contributions to Hospitals.** By Mr. W. Burdett-Coutts, M.P. - 191

**Bolingbroke House, Wandsworth Common** - 192

**The Doctor's Pulpit.**—A Day in Bed - 193

**Minor Aids to Medical Study.** - 194

**The Student's Column.**—Final Fellowship List - 194

**Physiology and its Uses.**—The Human Skull - 195

**Notes and News** - 196

**Everybody's Page** - 198

**Annotations.**—The Decay of Family Feeling, etc. - 199

**Round About the Asylums.** VIII. By a Medical Superintendent - 200

**An Ishmaelite.** By Leslie Keith, Author of "The Chilcotes," "East and West," etc. - 201

**The Dispensers' Column** - 202

**The "Universal Review" and Our Critics** - 203

**Frederick III., Emperor and King** - 203

**Amusements with Profit for all Ages.**—For Men and Women.—Children's Corner.—Puzzles, etc. - 204

**EXTRA SUPPLEMENT.—THE NURSING MIRROR.**

En Passant.—Lady Guardians.—Progress in Paris.—Sectarian Systems of Nursing.—Preachers on Nurses.—Croydon Palace.—Hospital Outlines.—Patients and Nurses - xlv
Notes from America.—"Life's Work Well Done" - xlvi
Original Articles.—A Nurses' Provident Fund - xlvi
The Nurses' Bookshelf.—Hospital Sisters.—Vacancies - xlvii
Everybody's Opinion.—The Pay of Private Nurses.—A Brave Hospital Nurse.—A Practical Experience.—Sir Frederick Leighton's Counsel - xlviii
Appointments.—Notes and News - xlviii

